# Provider Perspectives on the Impact of the COVID-19 Pandemic on Newborn Screening

**DOI:** 10.3390/ijns7030038

**Published:** 2021-07-07

**Authors:** Jessica I. Gold, Ian M. Campbell, Can Ficicioglu

**Affiliations:** 1 Division of Human Genetics, Department of Pediatrics, The Children’s Hospital of Philadelphia, Philadelphia, PA 19104, USA; campbellim@chop.edu; 2 Division of Human Genetics/Metabolism, Children’s Hospital of Philadelphia, Perelman School of Medicine at the University of Pennsylvania, Philadelphia, PA 19104, USA; ficicioglu@email.chop.edu

**Keywords:** newborn screening, telemedicine, COVID-19 pandemic, telegenetics

## Abstract

The onset of the COVID-19 pandemic caused significant changes in healthcare delivery. Telemedicine rapidly and unexpectedly became the primary vehicle for ambulatory management. As newborn screen (NBS) referrals require varying levels of acuity, whether telemedicine could be used as a safe and effective medium to return these results were unknown. We sent an online survey to metabolism providers internationally to investigate triage differences of abnormal NBS results during the COVID-19 pandemic. The survey compared personal practice for the periods of March–June 2019 and March–June 2020. Responses were received from 44 providers practicing in 8 countries. Nearly all (93%) practiced in areas of widespread SARS-COV-2 community transmission during spring 2020. There was a significant expansion of telemedicine use for NBS referrals at the onset of the COVID-19 pandemic (OR: 12, 95% CI: 3.66–39.3, *p* < 0.0001). Telehealth primarily replaced in-person ambulatory metabolism visits. The increased frequency of virtual care was similar across NBS analytes. Providers found telehealth for NBS referral equally efficacious to in-person care. Institutional patient surveys showed no difference in satisfaction with provider communication, provider empathy, or appointment logistics. Our survey was limited by unprecedented disruption in healthcare delivery, necessitating further validation of telegenetics for NBS in the post-pandemic era. Nevertheless, our findings demonstrate that telemedicine is potentially a viable and practical tool for triaging abnormal NBS results.

## 1. Introduction

The COVID-19 pandemic has created seismic shifts in our practice of healthcare. Urgently and unexpectedly, in-person visits were canceled, and providers navigated new platforms for telemedicine. Metabolism providers quickly learned that the age-old advice “there is no perfect time to have a baby” still applied during a global pandemic. During the onset of COVID-19, there was also no ideal way to manage newborn screen (NBS) referrals.

A delicate balance emerged between preventing exposure and urgently evaluating at-risk newborns. Prior to COVID-19, telemedicine had been promoted as a solution for the geographical limits of genetics providers [1,2,3]. Telegenetics programs in New Zealand and through the Western States Regional Genetic Network preceded the pandemic [4,5]. Increased utilization of telegenetics may help address workforce needs, especially in medical genetics “deserts” [1,6]. However, there is limited knowledge on telemedicine’s utility in triaging NBS results.

Our aim was to determine if metabolism providers engaged in telemedicine for NBS referrals during the initial months of the COVID-19 pandemic. We sought to identify differences in provider triage for abnormal NBS results during the pandemic. Lastly, we elicited provider and patient attitudes towards telemedicine to gauge its efficacy and likely use in the future.

## 2. Methods

### 2.1. Survey Instrument

The online survey was created to assess the use of telemedicine in the referral process for NBS during the initial months, March 2020–June 2020, of the COVID-19 pandemic (Figure S1). The survey contained 69 multiple-choice questions and 4 open-ended questions. When applicable, questions with “other” answer choices had an opportunity for free-text entry.

### 2.2. Survey Distribution

The survey was distributed via the Metab-L list server, an international mailing list for clinical care of inherited metabolic disorders. An email was sent containing a short invitation to complete the survey and a direct link with the survey’s REDCap URL. The email announcement was resent a total of 2 times at two-week intervals between July 2020 and August 2021.

### 2.3. Patient Satisfaction Scores

The Press Ganey patient survey database for the Children’s Hospital of Philadelphia (CHOP) was accessed on 16 April 2021. Patient surveys were filtered by age and the International Classification of Disease (ICD) code to identify newborn screen referrals. Surveys completed for visits between April 2019–July 2019 (all in-person visits) and visits between April 2020–July 2020 (all telehealth visits) were analyzed.

### 2.4. Statistical Analysis

Survey responses were analyzed using descriptive statistics. We used an ordinal mixed-effects model to analyze the change in practice before and after the pandemic. Our model included random effects for providers and their response to the pandemic. It included fixed effects for the pandemic, the analyte, and an interaction between the pandemic and the analyte. Results were reported as odds ratios (OR) with 95% confidence intervals. For categorical variables, the Chi-squared test or Fisher’s exact test were used, as appropriate. Two-sided *p*-values less than 0.05 were considered statistically significant. All analyses were conducted using the R statistical programing language.

## 3. Results

Our survey was completed by 44 respondents from 8 countries (Table 1). The majority of respondents were from the United States (75%) and represented diverse geographic areas. Over 93% of respondents practiced in regions with community transmission of SARS-COV-2 during the survey’s time period [7]. Thirteen respondents (29.5%) reported an institutional change in the NBS screen referral process during the initial months of the COVID-19 pandemic. These providers primarily practiced in the United States. 

**Figure 1:**
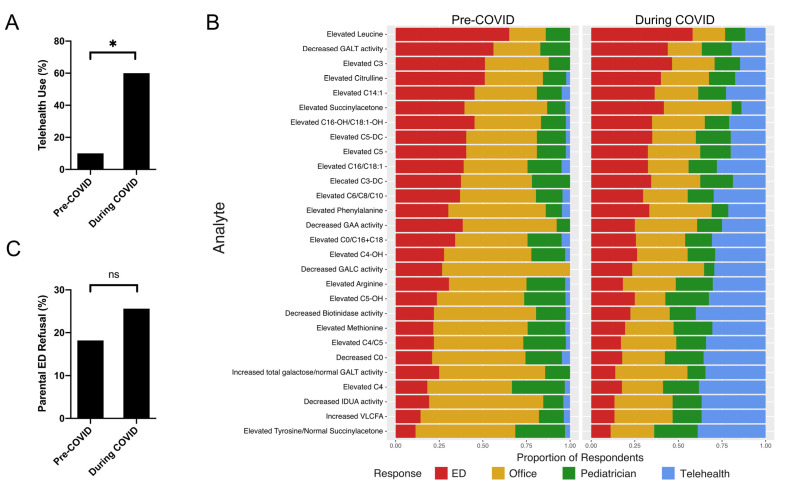
COVID-19 led to an increase in telehealth use for NBS referrals. (**A**) Use of telehealth for NBS follow-up significantly increased during March–June 2020, corresponding with the first wave of COVID-19. * *p* < 0.0001 (**B**) Respondents reported their referral pattern for NBS analytes pre-pandemic (**left**) and during the pandemic (**right**). Telehealth use increased for all analytes and primarily replaced in-person visits to metabolism clinic. (**C**) Providers did not report any significant difference in parental refusal to present to the Emergency Department (ED) for NBS follow-up. *p* = 0.41. ED: Emergency Department; ns: non-significant.

Respondents reported significant expansion of telemedicine use for NBS triage (Figure 1A). During the COVID-19 pandemic, 60% of providers used telemedicine for NBS follow-up compared to 9% before the pandemic (OR 12, 95% CI 3.66–39.3, *p* < 0.0001). Prior to the COVID-19 pandemic, NBS triage via telemedicine rarely occurred (Figure 1B). Only two respondents reported regular use. The onset of the COVID-19 pandemic led to significantly greater utilization of telemedicine for nearly all NBS analytes. The utilization of telemedicine in the face of COVID-19 was generally inversely proportional to the pre-pandemic acuity of each analyte. However, elevated succinylacetone and elevated phenylalanine were two biomarkers that resulted in unexpectedly high rates of in-person triage during the pandemic (succinylacetone, *p* = 0.029; phenylalanine, *p* = 0.043). 

**Figure 2:**
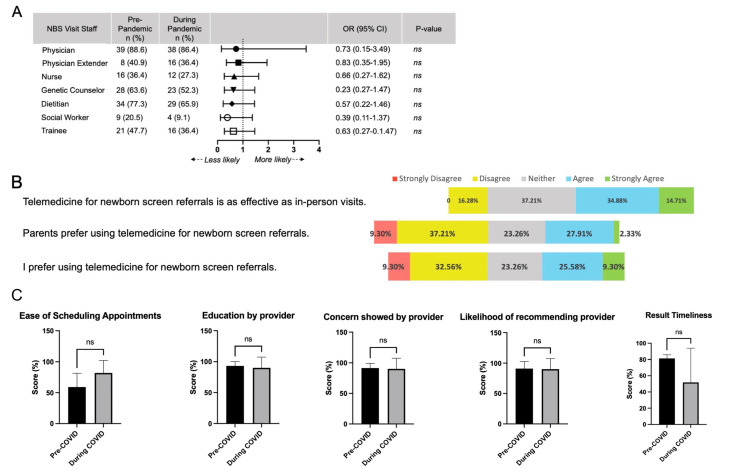
Telehealth for NBS referral is non-inferior to in-person clinic visits. (**A**) Odds ratios were calculated for staffing of NBS referrals prior to and during the pandemic. There was no significant different in staff presence during March-June 2020. (**B**) Provider responses recorded on a 5-point Likert scale regarding opinion on using telehealth during the COVID-19 pandemic. (**C**) Scores on Press Ganey patient surveys showed no difference in appointment logistics, provider communication, provider empathy, likelihood of recommending provider, or timeliness of receiving results. ns: non-significant.

Telehealth appointments primarily replaced in-person ambulatory visits to metabolism providers (Figure 1B). Triage for immediate assessment in an emergency department (ED) occurred at similar rates during the pandemic compared to the prior year. Respondents reported a slight increase in parental refusal for ED triage during the pandemic, but it was not significant (OR: 1.55, 95% CI: 0.55–4.3, *p* = 0.41) (Figure 1C). Clinical staffing for NBS follow-up was similar during the initial months of the COVID-19 pandemic (Figure 2A). There was no significant difference in visit personnel for any ancillary staff or trainees. This was consistent for both in-person and remote NBS visits. One respondent noted that telemedicine visits with ancillary staff might occur asynchronously instead of one coordinated visit with all participants.

Overall, respondents reported equivocal attitudes for the use of telemedicine for NBS follow-up. Nearly 50% agreed that “telemedicine for NBS referrals is as effective as in-person visits” (Figure 2B). Only 16.3% disagreed, indicating that remote NBS follow-up had comparable efficacy. Respondents were split on parental and personal preference for NBS-related telehealth. Only 30% of providers felt that parents preferred remote visits to in-person visits, while 35% of providers personally preferred telehealth themselves. While telehealth may have equal efficacy, nearly 50% indicated that telemedicine was an unfavorable way to conduct NBS triage.

Assessment of patient satisfaction with telemedicine was completed by comparing Press Ganey patient survey results from April–July 2019 and April–July 2020. All NBS referrals at CHOP were conducted via telemedicine during these months, while all NBS referrals occurred via in-person visits prior to March 2020. There was no significant difference in visit logistics—including ease of contacting the office and scheduling an appointment (Figure 2C). Providers received equivalent scores in education and empathy. Patients were equally likely to recommend providers seen in-person and virtually. Patients also reported no significant difference in the timeliness of receiving results.

## 4. Discussion

Telemedicine use in clinical genetics has attracted growing interest over the last decade [3]. The COVID-19 pandemic led to an unexpected expansion of telegenetics, leading to challenges in the diagnosis and management of inherited metabolic disorders (IMD) [8,9,10]. Our survey is the first to address provider perspective on telemedicine utility for the triage of NBS results. There was a significant pivot to telehealth following the onset of the COVID-19 pandemic. Prior to the pandemic, only two respondents regularly used remote visits for any NBS follow-up. In contrast, during the pandemic, many metabolic providers relied on telemedicine to manage NBS referrals. Telemedicine mainly replaced in-person visits to metabolism clinics. Urgent ED referral and primary assessment by a pediatrician occurred at the same rate pre-pandemic and during the pandemic. In general, telemedicine replaced in-person ambulatory evaluation at similar rates across NBS analytes. However, our analysis identified an unexpected preference for higher urgency evaluation for elevated succinylacetone and elevated phenylalanine despite the pandemic. Overall, the onset of the COVID-19 pandemic did not affect the respondent’s level of acuity for triaging abnormal NBS results. For example, elevations in C3 led to the same frequency of ED evaluations pre-COVID-19 and during the pandemic (OR: 0.75, 95% CI: 0.33–1.71, *p* = 0.49).

Providers reported similar advantages and disadvantages to telehealth use for NBS (Table 2). Greater convenience for families was perceived by providers as the leading advantage for using telehealth in NBS referrals. Many respondents were able to schedule telehealth appointments quickly, frequently occurring on the same day the NBS resulted. Fast turn-around time likely alleviates parental anxiety about the meaning of an abnormal NBS. Telemedicine was perceived to be more comfortable for families and decreased travel-related stress. It also allowed evaluation for families currently in quarantine or who were concerned about the risk of COVID-19 exposure. Better use of resources was also reported by 15% of respondents, especially when triaging non-acute conditions. Care coordination for remote visits was equivalent to remote visits, potentially leading to overall cost-savings (Figure 2A).

Difficulty arranging confirmatory laboratory testing was the main disadvantage to telemedicine for NBS (Table 2). Outside commercial laboratories may have less experience with venipuncture on neonates, leading to repeated attempts, poor quality specimens, and increased turn-around time. Our survey did not assess if commercial lab use led to any adverse sequelae due to diagnostic delays, but this metric should be included in future evaluations of telehealth use for NBS triage. Reliance on commercial laboratory service impacts business for the academic laboratories. The clinical volume for our biochemical laboratory decreased over 60% during our survey period compared to the prior three months. Diminished volume led to a significant financial loss for the biochemical laboratory. This reduced volume coincided with the complete transition to telemedicine for NBS referrals and routine outpatient follow-up. While our laboratory volumes have rebounded, they have not yet returned to pre-pandemic levels, likely due to the continued use of telehealth. Fully integrating telehealth into NBS referrals and metabolic medicine may threaten the viability of academic biochemical laboratories. 

Many respondents missed the physicality of in-person visits. Inability to perform a newborn physical exam was the main reported disadvantage for telehealth. Several respondents did wonder if a physical exam is truly necessary. One asked: “What do exams really contribute for most infants? I think we probably do not need them for most newborns”. Respondents were also concerned about building therapeutic relationships remotely. Nearly 40% of respondents did not feel confident about providing reassurance on telehealth platforms. They noted greater challenges in responding to non-verbal cues, which further complicates disclosure of difficult diagnoses. Patient satisfaction surveys do not mirror this provider’s concern. Patients scored provider-led education and empathy similarly for telehealth and in-person visits (Figure 2C). Lastly, connectivity remains problematic, especially in remote areas with limited internet access [11]. Audio-only visits compound the challenges of telehealth, further limiting the physical exam and relationship-building.

As in all surveys, our data is limited by self-selection bias. Survey completion may have occurred more frequently by providers in areas with higher COVID-19 incidence rates, leading to overestimation of telehealth expansion. Only three respondents practiced in areas without sustained community transmission. Respondents practicing in the Northeastern United States were most likely to have an institutional response to the pandemic, likely related to the high incidence rate [7]. Responses were only received from clinicians in developed countries, limiting the generalizability of our results. Access to technology significantly limits telemedicine’s viability. Future studies should address if gaps in connectivity hinders the expansion of telegenetics beyond developed nations. Regardless of the impetus for telemedicine use, the COVID-19 pandemic proved an excellent case study in conducting NBS referrals remotely. 

Our survey examined provider response to NBS referrals during the pandemic. To fully understand this new clinical circumstance, it is imperative to further explore the attitudes of families referred for remote management of NBS. Telemedicine has previously been shown to have high patient satisfaction for pediatrics visits, cancer genetics counseling, and presymptomatic genetic counseling [12,13,14,15]. Similar positive sentiment will likely be seen for NBS referrals, although tempered slightly by the need to obtain confirmatory laboratories. Analysis of CHOP’s patient surveys indeed shows that patients are equally satisfied with telemedicine compared to in-person visits. However, these surveys were collected at a time of unprecedented shifts in medical and social practices. Family satisfaction with telegenetics may be artificially inflated due to the extraordinary circumstances of the COVID-19 pandemic. Future evaluations of patient opinion on telemedicine should occur after regular in-person ambulatory visits resume. Our institution currently offers parents a choice between telemedicine and in-person visits for NBS follow-up, creating ideal comparison groups More investigation is also needed to define the specific benefits of telehealth in a diverse patient population from multiple medical systems. Examining the patient and family experience with telehealth during a period of relative calm will aid its use moving forward.

In the United States, the future viability of telemedicine will depend heavily on bureaucratic barriers. Each state issues medical licenses independently for practices within their state. Prior to COVID-19, physicians were required to be licensed in the state where the patient is located [16]. This requirement was waived at the onset of the pandemic, allowing clinicians to provide medical services over a greater geographic area [17]. Restrictions on insurance coverage and reimbursement were also lifted in spring 2020 to promote telemedicine use [18,19]. Uncertainty about continued provisions may prevent the widespread adoption of telehealth for NBS. More evidence on the benefits of telegenetics may encourage federal-level solutions to medical licensure and reimbursement patterns. 

The COVID-19 pandemic prompted unparalleled innovation in healthcare delivery. We cannot predict whether telemedicine for NBS will persist beyond this year of extraordinary upheaval. Currently, providers are more seasoned at conducting visits remotely and may have stronger opinions on their efficacy. Leading virtual discussions on complex topics is now common. It will be necessary to reassess telemedicine’s utility for returning NBS in a post-pandemic world from the perspective of both clinicians and families. Repeating this survey in 3–5 years will allow us to track the longevity of the unexpected expansion into telehealth for NBS. Future studies should also explore how disparities in access to technology affect telegenetics efficacy, focusing on respondents from developing nations. It is critical to include families from diverse backgrounds in these studies to fully assess the feasibility of telemedicine for NBS. 

## 5. Conclusions

Telegenetics rose to prominence during the COVID-19 pandemic. It is a feasible solution for limitations in workforce numbers and geographical distributions of clinical geneticists. Overall, our survey showed that telemedicine for NBS referrals was equally effective to in-person visits for metabolic providers during the COVID-19 pandemic. Greater familial convenience, including decreased need to travel with a newborn and quicker scheduling being the leading perceived advantages for telehealth use. Future work is needed to assess family attitudes toward telemedicine use for NBS referrals, especially as healthcare delivery returns to a post-pandemic status quo. While the initial response to the COVID-19 pandemic occurred emergently, it has imparted lessons on telehealth utilization in biochemical genetics. Family preference, administrative barriers, and provider proclivity will influence the future of telegenetics use for NBS.

## Figures and Tables

**Table 1:** Respondent demographics.

Location	Total, *n* (%)	Cumulative COVID-19 Incidence Rate (through 12 July 2020)	WHO-Designated Pattern of Spread	Institutions with COVID-Related Protocols, *n*
United States	33 (76.7)	2,573,393	Community Transmission	11
Australia	1 (2.3)	7834	Clusters of Cases	1
Canada	3 (6.9)	103,918	Community Transmission	-
Germany	1 (2.3)	194,725	Clusters of Cases	-
Italy	1 (2.3)	240,578	Community Transmission	-
New Zealand	1 (2.3)	1178	Clusters of Cases	-
Switzerland	1 (2.3)	31,631	Community Transmission	-
United Kingdom	2 (4.7)	31,658	Community Transmission	1
No location indicated	1 (2.3)	-	-	-

**Table 2:** Clinician perceptions of telehealth for NBS during the COVID-19 pandemic.

	Clinicians Endorsing (%)
**Advantages**	
Greater convenience for families	67.5
Easier to schedule	65.0
More comfortable for families	20.0
Wider catchment area	12.5
Avoid COVID exposure to newborn	10.0
Helpful to triage non-acute conditions	10.0
Saves resources	5.0
More frequent follow-up	2.5
Allows evaluation for quarantining families	2.5
**Disadvantages**	
Physical exam very limited	47.6
Limited ability to provide reassurance	38.1
Difficult to arrange testing	38.1
Challenging to disclose difficult diagnoses	21.4
Poor relationship building with family	14.3
Concern about outside labs	14.3
Difficulty coordinating multidisciplinary care	9.5
Increased lab turn-around time	9.5
Inability to respond to non-verbal cues	9.5
Cannot start dietary treatment at visit	7.1

## Data Availability

Data are stored in the CHOP REDCap database and are available upon request to the corresponding author.

## Supplementary Materials

The following are available online at https://www.mdpi.com/article/10.3390/ijns7030038/s1, Figure S1: Paper version of the online survey tool including prompts for conditional branching (skip logic).
