# Supplementary material for: Provider Perspectives on the Impact of the COVID-19 Pandemic on Newborn Screening
Source: Int J Neonatal Screen. 2021 Jul 7;7(3):38. doi: 10.3390/ijns7030038 (PMC8293089; doi:10.3390/ijns7030038)
Supplement: Supplementary file 1 [file IJNS-07-00038-s001.zip › IJNS-1233280-SI.pdf]

SUPPLEMENTAL INFORMATION

Fig S1. Paper version of the online survey tool including prompts for conditional branching (skip logic).

We are examining how the COVID-19 pandemic and the increasing use of telehealth has affect your newborn screen practices. This **confidential online research survey** will take approximately 5-15 minutes to complete All collected data will be reported collectively in aggregate to ensure confidentiality.

Please complete the survey below.

1. Location (City, State or City, Country):
2. Has your institution's Newborn Screen referral process changed during the COVID-19 pandemic?
    a. Yes
    b. No

    If the answer is a. Yes → #3. If the answer is b. No → #4.

3. If yes, please explain how your NBS referral process differs:

Please select the option(s) that best reflects triage for each of the following dried blood spot (DBS) amino acid results PRIOR to COVID-19.

4. Elevated Arginine
    a. Immediate referral to care
    b. Referral for office visit
    c. Referral to pediatrician
    d. Telemedicine visit
    e. Other: ______________________

5. Elevated Citrulline
    a. Immediate referral to care
    b. Referral for office visit
    c. Referral to pediatrician
    d. Telemedicine visit
    e. Other: ______________________

6. Elevated Methionine
    a. Immediate referral to care
    b. Referral for office visit
    c. Referral to pediatrician
    d. Telemedicine visit
    e. Other: ______________________

7. Elevated Leucine
    a. Immediate referral to care
    b. Referral for office visit
    c. Referral to pediatrician
    d. Telemedicine visit
    e. Other: ______________________

8. Elevated Phenylalanine
    a. Immediate referral to care
    b. Referral for office visit
    c. Referral to pediatrician
    d. Telemedicine visit
    e. Other: ______________________

9. Elevated Tyrosine with normal succinylacetone
    a. Immediate referral to care
    b. Referral for office visit
    c. Referral to pediatrician
    d. Telemedicine visit
    e. Other: ______________________

10. Elevated Succinylacetone with normal or elevated tyrosine
    a. Immediate referral to care
    b. Referral for office visit
    c. Referral to pediatrician
    d. Telemedicine visit
    e. Other: ______________________

Please select the option(s) that best reflects triage for the following DBS results for fatty acid oxidation disorders PRIOR to COVID-19.

11. Decreased C0
    a. Immediate referral to care
    b. Referral for office visit
    c. Referral to pediatrician
    d. Telemedicine visit
    e. Other: ______________________

12. Elevated C0/C16+C18
    a. Immediate referral to care
    b. Referral for office visit
    c. Referral to pediatrician
    d. Telemedicine visit
    e. Other: ______________________

13. Elevated C16 and/or C18:1
    a. Immediate referral to care
    b. Referral for office visit
    c. Referral to pediatrician
    d. Telemedicine visit
    e. Other: ______________________

14. Elevated C4; C5
    a. Immediate referral to care
    b. Referral for office visit
    c. Referral to pediatrician
    d. Telemedicine visit
    e. Other: ______________________

15. Elevated C16-OH and C18:1-OH
    a. Immediate referral to care
    b. Referral for office visit
    c. Referral to pediatrician
    d. Telemedicine visit
    e. Other: ______________________

16. Elevated C8, C6, C10
    a. Immediate referral to care
    b. Referral for office visit
    c. Referral to pediatrician
    d. Telemedicine visit
    e. Other: ______________________

17. Elevated C4-OH
    a. Immediate referral to care
    b. Referral for office visit
    c. Referral to pediatrician
    d. Telemedicine visit
    e. Other: ______________________

18. Elevated C4
    a. Immediate referral to care
    b. Referral for office visit
    c. Referral to pediatrician
    d. Telemedicine visit
    e. Other: ______________________

19. Elevated C14:1
    a. Immediate referral to care
    b. Referral for office visit

c. Referral to pediatrician
d. Telemedicine visit
e. Other: ______________________

Please select the option(s) that best reflects triage for the following DBS results for organic acidemias PRIOR to COVID-19.

20. Elevated C5-OH
    a. Immediate referral to care
    b. Referral for office visit
    c. Referral to pediatrician
    d. Telemedicine visit
    e. Other: ______________________

21. Elevated C5-DC
    a. Immediate referral to care
    b. Referral for office visit
    c. Referral to pediatrician
    d. Telemedicine visit
    e. Other: ______________________

22. Elevated C5
    a. Immediate referral to care
    b. Referral for office visit
    c. Referral to pediatrician
    d. Telemedicine visit
    e. Other: ______________________

23. Elevated C3-DC
    a. Immediate referral to care
    b. Referral for office visit
    c. Referral to pediatrician
    d. Telemedicine visit
    e. Other: _____________________

24. Elevated C3
    a. Immediate referral to care
    b. Referral for office visit
    c. Referral to pediatrician
    d. Telemedicine visit
    e. Other: ______________________

Please select the option(s) that best reflects triage for the following DBW results PRIOR to COVID-19.

25. Absent or reducted GALT

a. Immediate referral to care
b. Referral for office visit
c. Referral to pediatrician
d. Telemedicine visit
e. Other: ______________________

26. Increased total galactose with normal GALT
    a. Immediate referral to care
    b. Referral for office visit
    c. Referral to pediatrician
    d. Telemedicine visit
    e. Other: ______________________

27. Biotinidase deficiency
    a. Immediate referral to care
    b. Referral for office visit
    c. Referral to pediatrician
    d. Telemedicine visit
    e. Other: ______________________

Please select the option(s) that best reflects triage for the following DBS results for lysosomal storage disease PRIOR to COVID-19.

28. Krabbe disease
    a. Immediate referral to care
    b. Referral for office visit
    c. Referral to pediatrician
    d. Telemedicine visit
    e. Other: ______________________
29. MPS I
    a. Immediate referral to care
    b. Referral for office visit
    c. Referral to pediatrician
    d. Telemedicine visit
    e. Other: ______________________

30. Pompe Disease
    a. Immediate referral to care
    b. Referral for office visit
    c. Referral to pediatrician
    d. Telemedicine visit
    e. Other: ______________________

31. X-ALD
    a. Immediate referral to care
    b. Referral for office visit

c. Referral to pediatrician
d. Telemedicine visit
e. Other: ______________________

Please select the option(s) that best reflects triage for each of the following dried blood spot amino acid results DURING COVID-19.

32. Elevated Arginine
    a. Immediate referral to care
    b. Referral for office visit
    c. Referral to pediatrician
    d. Telemedicine visit
    e. Other: ______________________

33. Elevated Citrulline
    a. Immediate referral to care
    b. Referral for office visit
    c. Referral to pediatrician
    d. Telemedicine visit
    e. Other: ______________________

34. Elevated Methionine
    a. Immediate referral to care
    b. Referral for office visit
    c. Referral to pediatrician
    d. Telemedicine visit
    e. Other: ______________________

35. Elevated Leucine
    a. Immediate referral to care
    b. Referral for office visit
    c. Referral to pediatrician
    d. Telemedicine visit
    e. Other: ______________________

36. Elevated Phenylalanine
    a. Immediate referral to care
    b. Referral for office visit
    c. Referral to pediatrician
    d. Telemedicine visit
    e. Other: ______________________

37. Elevated Tyrosine with normal succinylacetone
    a. Immediate referral to care
    b. Referral for office visit
    c. Referral to pediatrician

d. Telemedicine visit
e. Other: ______________________

38. Elevated Succinylacetone with normal or elevated tyrosine
    a. Immediate referral to care
    b. Referral for office visit
    c. Referral to pediatrician
    d. Telemedicine visit
    e. Other: ______________________

Please select the option(s) that best reflects triage for the following DBS results for fatty acid oxidation disorders DURING COVID-19.

39. Decreased C0
    a. Immediate referral to care
    b. Referral for office visit
    c. Referral to pediatrician
    d. Telemedicine visit
    e. Other: ______________________

40. Elevated C0/C16+C18
    a. Immediate referral to care
    b. Referral for office visit
    c. Referral to pediatrician
    d. Telemedicine visit
    e. Other: ______________________

41. Elevated C16 and/or C18:1
    a. Immediate referral to care
    b. Referral for office visit
    c. Referral to pediatrician
    d. Telemedicine visit
    e. Other: ______________________

42. Elevated C4; C5
    a. Immediate referral to care
    b. Referral for office visit
    c. Referral to pediatrician
    d. Telemedicine visit
    e. Other: ______________________

43. Elevated C16-OH and C18:1-OH
    a. Immediate referral to care
    b. Referral for office visit
    c. Referral to pediatrician

d. Telemedicine visit
e. Other: ______________________

44. Elevated C8, C6, C10
    a. Immediate referral to care
    b. Referral for office visit
    c. Referral to pediatrician
    d. Telemedicine visit
    e. Other: ______________________

45. Elevated C4-OH
    a. Immediate referral to care
    b. Referral for office visit
    c. Referral to pediatrician
    d. Telemedicine visit
    e. Other: ______________________

46. Elevated C4
    a. Immediate referral to care
    b. Referral for office visit
    c. Referral to pediatrician
    d. Telemedicine visit
    e. Other: ______________________

47. Elevated C14:1
    a. Immediate referral to care
    b. Referral for office visit
    c. Referral to pediatrician
    d. Telemedicine visit
    e. Other: ______________________

Please select the option(s) that best reflects triage for the following DBS results for organic acidemias DURING COVID-19.

48. Elevated C5-OH
    a. Immediate referral to care
    b. Referral for office visit
    c. Referral to pediatrician
    d. Telemedicine visit
    e. Other: ______________________

49. Elevated C5-DC
    a. Immediate referral to care
    b. Referral for office visit
    c. Referral to pediatrician
    d. Telemedicine visit

    e. Other: ______________________

50. Elevated C5
    a. Immediate referral to care
    b. Referral for office visit
    c. Referral to pediatrician
    d. Telemedicine visit
    e. Other: ______________________

51. Elevated C3-DC
    a. Immediate referral to care
    b. Referral for office visit
    c. Referral to pediatrician
    d. Telemedicine visit
    e. Other: _____________________

52. Elevated C3
    a. Immediate referral to care
    b. Referral for office visit
    c. Referral to pediatrician
    d. Telemedicine visit
    e. Other: ______________________

Please select the option(s) that best reflects triage for the following DBS results DURING COVID-19.

53. Absent or reducted GALT
    a. Immediate referral to care
    b. Referral for office visit
    c. Referral to pediatrician
    d. Telemedicine visit
    e. Other: ______________________

54. Increased total galactose with normal GALT
    a. Immediate referral to care
    b. Referral for office visit
    c. Referral to pediatrician
    d. Telemedicine visit
    e. Other: ______________________

55. Biotinidase deficiency
    a. Immediate referral to care
    b. Referral for office visit
    c. Referral to pediatrician
    d. Telemedicine visit
    e. Other: ______________________

Please select the option(s) that best reflects triage for the following DBS results for lysosomal storage disease DURING COVID-19.

56. Krabbe disease
    a. Immediate referral to care
    b. Referral for office visit
    c. Referral to pediatrician
    d. Telemedicine visit
    e. Other: ______________________
57. MPS I
    a. Immediate referral to care
    b. Referral for office visit
    c. Referral to pediatrician
    d. Telemedicine visit
    e. Other: ______________________

58. Pompe Disease
    a. Immediate referral to care
    b. Referral for office visit
    c. Referral to pediatrician
    d. Telemedicine visit
    e. Other: ______________________

59. X-ALD
    a. Immediate referral to care
    b. Referral for office visit
    c. Referral to pediatrician
    d. Telemedicine visit
    e. Other: ______________________

60. PRIOR to COVID-19, did any parents refuse referral to the emergency room?
    a. Yes
    b. No

    If the answer is a. Yes → #61. If the answer is b. No → #62.

61. In your recollection, PRIOR to COVID-19, how many times did parents refuse referral to the emergency room?
    a. Less than 5
    b. 5-10
    c. 11-20
    d. More than 20

62. DURING COVID-19, did any parents refuse referral to the emergency room?
    a. Yes

    b. No

    If the answer is a. Yes → #63. If the answer is b. No → #64.

63. In your recollection, DURING COVID-19, how many times did parents refuse referral to the emergency room?
    a. Less than 5
    b. 5-10
    c. 11-20
    d. More than 20

64. PRIOR to COVID-19, was your institution regularly using telemedicine for newborn screen visits?
    a. Yes
    b. No

65. DURING COVID-19, is your institution regularly using telemedicine for initial newborn screen visits?
    a. Yes
    b. No

66. PRIOR to COVID-19, which of the following professionals were routinely present during newborn screen referral visits?
    a. MD/DO
    b. NP/PA
    c. RN
    d. Genetic Counselor
    e. Dietitian
    f. Social Worker
    g. Trainee (fellow/resident/medical student)
    h. Other: ______________________

67. DURING COVID-19, which of the following professionals were routinely present during newborn screen referral visits?
    a. MD/DO
    b. NP/PA
    c. RN
    d. Genetic Counselor
    e. Dietitian
    f. Social Worker
    g. Trainee (fellow/resident/medical student)
    h. Other: ______________________

68. Newborn screen referrals are easier with telemedicine
    a. Strongly Disagree
    b. Disagree

c. Neither Agree nor Disagree
d. Agree
e. Strongly Agree

69. Telemedicine for newborn screen referrals is as effective as in-person visits.
    a. Strongly Disagree
    b. Disagree
    c. Neither Agree nor Disagree
    d. Agree
    e. Strongly Agree

70. Parents prefer using telemedicine for newborn screen referrals
    a. Strongly Disagree
    b. Disagree
    c. Neither Agree nor Disagree
    d. Agree
    e. Strongly Agree

71. I prefer using telemedicine for newborn screen referrals.
    a. Strongly Disagree
    b. Disagree
    c. Neither Agree nor Disagree
    d. Agree
    e. Strongly Agree

72. In your opinion, what are the advantages to using telemedicine for newborn screen referrals?

73. In your opinion, what are the disadvantages to using telemedicine for newborn screen referrals?
